# Association Between Chin-Tuck-Generated Force and Sarcopenia in Community-Dwelling Older Adults: A Cross-Sectional Study

**DOI:** 10.3390/healthcare14081018

**Published:** 2026-04-13

**Authors:** Naoto Kamide, Takeshi Murakami, Takuya Sawada, Masataka Ando, Miki Sakamoto

**Affiliations:** 1 School of Allied Health Sciences, Kitasato University, Kitazato 1-15-1, Minami-ku, Sagamihara 252-0373, Japan; takeshim@kitasato-u.ac.jp (T.M.); sawada.takuya2@kitasato-u.ac.jp (T.S.); m.ando@kitasato-u.ac.jp (M.A.); mikis@kitasato-u.ac.jp (M.S.); 2 Graduate School of Medical Sciences, Kitasato University, Kitazato 1-15-1, Minami-ku, Sagamihara 252-0373, Japan

**Keywords:** chin-tuck, older adults, sarcopenia, suprahyoid muscle, swallowing-related muscle, tongue pressure

## Abstract

**Background**: Although swallowing-related muscle function has been implicated in sarcopenia, the association between swallowing-related cervical muscle function and sarcopenia has not been thoroughly examined. The aim of this study was to investigate this association in community-dwelling older adults. **Methods**: This cross-sectional study included 390 community-dwelling adults aged ≥65 years. Sarcopenia was defined as the concurrent presence of low handgrip strength and low appendicular skeletal muscle mass. The force generated during the chin-tuck maneuver (chin-tuck force) was measured using a dynamometer to indicate swallowing-related cervical muscle function. Tongue pressure and oral diadochokinesis were measured as indicators of swallowing-related muscle function. Potential confounders included body mass index, comorbidities, number of medications, functional capacity, timed up-and-go test and trail-making test times. **Results**: In logistic regression analyses adjusted for age and sex, chin-tuck force was found to have a statistically significant association with sarcopenia; greater force correlated inversely with sarcopenia (odds ratio = 0.59, *p* < 0.001). Receiver operating characteristic curve analysis demonstrated acceptable discriminative ability of chin-tuck force for identifying sarcopenia (area under the curve (AUC) = 0.82, 95% confidence interval (CI): 0.72–0.90), which was significantly higher than that for tongue pressure (AUC = 0.62, 95% CI: 0.50–0.74; *p* < 0.01). **Conclusions**: Among swallowing-related muscle functions, reduced chin-tuck force may be associated with sarcopenia in older adults. Future studies should investigate targeted assessments and interventions focused on improving swallowing-related cervical muscle function as a potential strategy for sarcopenia prevention.

## 1. Introduction

Sarcopenia is defined as a progressive, generalized skeletal muscle disease characterized by declines in both muscle mass and muscle strength with advancing age [1]. It is recognized as a major geriatric risk factor and is associated with numerous adverse health outcomes, including increased risks of falls, fractures, reduced basic and instrumental activities of daily living (ADL and IADL), cognitive impairment, hospitalization, and mortality [2,3,4,5,6,7]. A meta-analysis estimated the global prevalence of sarcopenia to be approximately 10% in both men and women [8], indicating that it is a common age-related disease. Therefore, early detection and preventive interventions for sarcopenia are essential clinical priorities for healthcare professionals caring for older adults.

Multiple risk factors contribute to the development of sarcopenia. Physical inactivity and malnutrition are widely recognized modifiable factors, whereas acute and chronic wasting conditions caused by organ failure, inflammatory disease, malignancy, and endocrine disorders are also important contributors [9]. In particular, in community-dwelling older adults, adequate physical activity and nutritional intake are strongly recommended for preventing sarcopenia [10]. In addition, in the most recent consensus update by the Asian Working Group for Sarcopenia (AWGS), swallowing dysfunction (dysphagia) has also been mentioned as a risk factor [11] and is a common geriatric syndrome among community-dwelling older adults [12], with a systematic review reporting a prevalence of 20.35% [13].

Recently, increasing attention has been directed toward the relationship between sarcopenia and swallowing dysfunction, highlighted through the concept of sarcopenic dysphagia, which is characterized by reduced muscle mass and strength in both whole-body musculature and swallowing-related muscles [14]. Although longitudinal evidence remains limited, previous systematic reviews and meta-analyses have shown clear associations between sarcopenia and impaired swallowing-related muscle function in community-dwelling older people [15]. Accordingly, swallowing-related muscle weakness may represent an important risk factor for sarcopenia in community-dwelling older adults.

Regarding the swallowing-related muscles and sarcopenia, several studies have reported associations between tongue pressure, which is widely used as an indicator of swallowing muscle strength, and sarcopenia [15,16,17,18,19]. However, among swallowing-related cervical muscles, the suprahyoid muscle group, which plays a crucial role in hyoid elevation and opening of the pharyngoesophageal segment, may be particularly important. Age-related changes in the geniohyoid muscle, including reduced muscle mass and increased intramuscular fat, have been documented [20,21,22]. Moreover, targeted interventions, such as the Shaker exercise and chin-tuck against resistance training, have demonstrated improvements in swallowing function by strengthening the suprahyoid muscles [23,24,25]. Thus, assessment of suprahyoid muscle function may be essential for understanding swallowing-related risk factors for sarcopenia. Although other cervical flexor muscles may also contribute to the generated force, the chin-tuck maneuver is known to strongly activate the suprahyoid muscles [25].

Despite these findings, research examining the relationship between sarcopenia and swallowing-related cervical muscle function, including the suprahyoid muscles, remains limited due to a lack of suitable assessment methods. To address this gap, we developed a device designed to measure the force generated during the chin-tuck maneuver (chin-tuck force), which reflects swallowing-related cervical muscle function, including the activity of the suprahyoid muscles. The reliability and validity of this device have been previously confirmed [26]. Although previous studies have assessed tongue pressure and ultrasound-based morphology of the suprahyoid muscles, few have directly examined the relationship between swallowing-related cervical muscle function and sarcopenia in community-dwelling older adults. Therefore, the aim of this cross-sectional study was twofold: first, to clarify the relationship between sarcopenia and force generated during the chin-tuck maneuver as an indicator of swallowing-related cervical muscle function, and second, to investigate the discriminative ability of chin-tuck force for sarcopenia in community-dwelling older adults. By further elucidating the association between swallowing-related muscle function and overall muscle health, this study may contribute to identifying potential therapeutic targets for sarcopenia prevention and management.

## 2. Materials and Methods

### 2.1. Participants

This cross-sectional observational study included 390 community-dwelling older adults aged 65 years and above who were living independently in Sagamihara City, Japan. Participants were recruited through advertisements in local newspapers and posters displayed in public facilities. Data for this study was collected between March and September of 2025. Individuals were eligible to participate if they were aged 65 years or older, had no restrictions on oral food intake, as verified by the Functional Oral Intake Scale [27], and were independent in their activities of daily living. Individuals were excluded if they were certified as needing support or care level by the Japanese long-term care insurance system, had neck pain or neurological symptoms, such as numbness or weakness associated with cervical myelopathy or cervical disc herniation, had neurodegenerative diseases, such as Parkinson’s disease or dementia, if they were institutionalized, or if they had a pacemaker. These exclusion criteria were confirmed through a self-reported questionnaire and a face-to-face interview conducted by trained researchers.

Sample size was calculated using a previously reported mean difference in tongue pressure of 5.4 kPa, and a standard deviation of 7.4 kPa between individuals with and without sarcopenia [15], assuming a sarcopenia prevalence of 6.2% [28], an alpha level of 0.05, and a statistical power of 0.80. Based on these parameters, the required sample size was estimated to be 272 participants.

The study received ethical approval from the Institutional Review Board of the School of Allied Health Sciences at Kitasato University (Approval No. 2023-008). The purpose and content of the study were explained to the participants both orally and in a written document. Participants were also informed of their right to withdraw and how incidental findings would be handled. Written informed consent was obtained from all participants.

### 2.2. Assessment of Swallowing-Related Muscle Function and Oral Function

**Figure 1:**
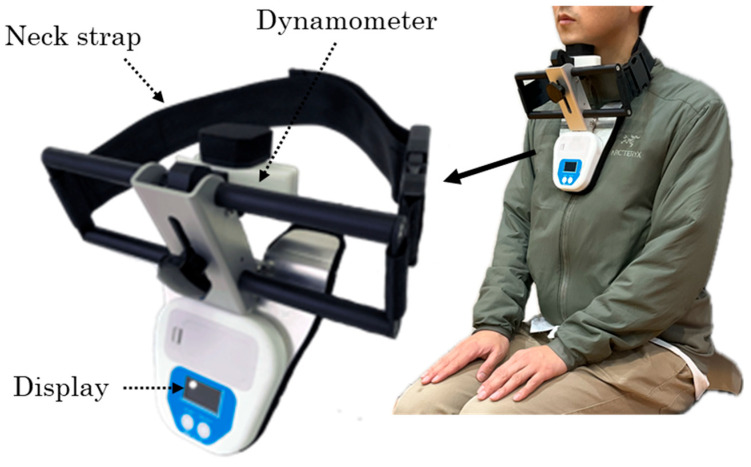
Device for measuring force generated during chin-tuck.

Swallowing-related muscle function was evaluated through measurement of chin-tuck force, tongue pressure, and oral diadochokinesis (ODK), and oral function was assessed using an established oral frailty scale. Chin-tuck force was measured as the isometric maximum force generated during a chin-tuck maneuver using a dynamometer (Neckforce, T.K.K. 3359, SANKA Co., Ltd., Niigata, Japan) (Figure 1). The device was positioned with the participant’s neck in the neutral position, and measurements were taken while the participant sat on a standard armless chair with their back resting against the backrest. Participants were instructed to tuck their chin maximally toward the manubrium sterni and maintain the isometric contraction for five seconds, during which the isometric maximum force [kg] was recorded. Two trials were performed with a 30 s rest period between trials, and the mean value was used for analysis. Previous studies have demonstrated high reliability of this method, with intraclass correlation coefficients ranging from 0.82 to 0.89, supporting its validity for assessing swallowing-related muscle strength [26]. Before the measurement, participants performed one practice trial to familiarize themselves with the chin-tuck maneuver. Participants were instructed to immediately inform the examiner if they experienced any pain or discomfort during the procedure, in which case the measurement would be discontinued.

Tongue pressure was measured using a standardized device (TPM-02, JMS Co., Ltd., Hiroshima, Japan) following previously established procedures [29]. Three measurements were obtained with a 30 s interval between trials, and the average value was used for analysis. ODK was assessed using a dedicated measurement device (T.K.K. 3351, SANKA Co., Ltd., Niigata, Japan). Participants were instructed to separately repeat the syllables “pa,” “ta,” and “ka” as rapidly as possible, for 5 s each, and the number of repetitions per second for each syllable was recorded. Oral function was further assessed using the Oral Frailty Five-Item Checklist (OF-5), which consists of self-reported items addressing fewer teeth, chewing difficulty, swallowing difficulty, dry mouth, and reduced articulatory motor skills [30]. Possible total scores range from 0 to 5. Scores of two or higher indicate oral frailty and have been confirmed as valid for Japanese community-dwelling older adults [30].

### 2.3. Sarcopenia

Sarcopenia was defined according to the AWGS 2025 criteria [11]. Muscle strength was assessed using handgrip strength measured with a Smedley-type dynamometer (T.K.K. 5401, SANKA Co., Ltd.). Values below 28 kg for men and 18 kg for women were classified as low muscle strength. Appendicular skeletal muscle mass (ASM) was measured using bioelectrical impedance analysis (InBody 430; InBody Japan Inc., Tokyo, Japan), and skeletal muscle mass index (SMI) was calculated by dividing ASM by height (in meters) squared (kg/m^2^). Low muscle mass was defined as an SMI below 7.0 kg/m^2^ for men and below 5.7 kg/m^2^ for women. In accordance with the AWGS 2025 criteria, participants in this study were classified as having sarcopenia only if they exhibited both low muscle strength and low muscle mass. Those with only one of these conditions were not categorized as ‘possible’ or ‘at risk’, but were included in the non-sarcopenia group. According to the updated AWGS 2025 framework, physical performance is considered an outcome rather than a diagnostic component; therefore, gait speed and other physical performance measures were excluded from the diagnostic definition in this study.

### 2.4. Potential Confounding Factors

Potential confounders were assessed using a combination of self-reported questionnaires and objective measures. The questionnaire collected information on age, physician-diagnosed comorbidities, number of prescribed medications, living arrangement (living alone or not), depressive symptoms, and dietary protein intake. Polypharmacy was defined as the use of six or more medications. Functional capacity was assessed using the Tokyo Metropolitan Institute of Gerontology Index of Competence (TMIG-IC), which evaluates higher-level functional abilities such as IADL and social roles, and ranges from 0 to 13 points, with higher scores indicating better functional capacity [31]. Depressive symptoms were evaluated using the five-item Geriatric Depression Scale, with scores of two or higher indicating the presence of depressive symptoms [32]. Protein intake status was assessed using consumption markers for protein intake from the Mini Nutritional Assessment^®^, including daily intake of dairy products, consumption of legumes or eggs at least twice weekly, and daily intake of meat or fish [33].

Mobility was assessed using the Timed Up and Go (TUG) test performed at the fastest walking speed, and cognitive function was assessed using the Trail Making Test (TMT) Parts A and B. Body mass index (BMI) was calculated from measured height and weight.

### 2.5. Statistical Analysis

Initially, the associations between the presence of sarcopenia and swallowing-related muscle functions, oral function, and all potential confounding factors were examined using *t*-tests or Fisher’s exact tests.

Logistic regression analyses adjusted for age and sex were conducted to examine the association between each swallowing-related muscle function measure and sarcopenia. Each measure was analyzed in a separate model. As an exploratory analysis, additional logistic regression models were constructed with further adjustment for potential confounding factors. Each measure was also analyzed in a separate model. These confounders were selected based on their clinical relevance and their association with sarcopenia in the univariate analysis. Exploratory analyses were conducted to provide additional context, but they should be interpreted cautiously due to the limited number of events. Finally, collinearity was considered using the variance inflation factor.

Receiver operating characteristic (ROC) curve analysis was performed to evaluate the discriminatory ability of each swallowing-related muscle function for identifying sarcopenia, and an area under the curve (AUC) ≥ 0.7 was considered as acceptable discrimination [34]. The 95% confidence intervals for the AUCs were estimated using bootstrap resampling with 1000 iterations to account for potential optimism due to the limited number of events. Furthermore, comparisons of discriminatory ability among swallowing-related muscle functions were conducted using a test for AUC by means of the bootstrap method with 1000 iterations, net reclassification improvement (NRI), and integrated discrimination improvement (IDI). It has been argued that the test for AUCs has limited ability to detect changes in discriminative ability. This study therefore calculated the NRI and IDI to complement the test for AUCs and provide additional information on improvements in risk classification and discrimination. The significance level was set at 5%, and all statistical analyses were performed using R version 4.2.2 (R Foundation for Statistical Computing, Vienna, Austria). The “pROC” package was used for ROC curve analysis.

## 3. Results

### 3.1. Characteristics of the Participants

Descriptive statistics for all collected variables are presented in Table 1. Regarding sarcopenia, 35 participants had low muscle strength and 104 had low muscle mass; consequently, 21 participants (5.4%) were diagnosed with sarcopenia. Among medical conditions, hypertension was the most frequently reported comorbidity, whereas other diseases—including diabetes mellitus, stroke, kidney disease, respiratory disease, and heart disease—were relatively uncommon. There was no statistically significant association between comorbidities and polypharmacy and the presence of sarcopenia. No statistically significant associations were found between sarcopenia and living arrangement (living alone), depressive symptoms, TMIG-IC score, or dietary protein intake. In contrast, age, BMI, TUG time, and TMT Part B time differed significantly by sarcopenia status. Participants with sarcopenia were older, had lower BMI, and demonstrated longer TUG and TMT Part B times compared with those without sarcopenia.

### 3.2. Swallowing-Related Muscle Function, Oral Function, and Sarcopenia

For chin-tuck force, none of the participants discontinued the measurement due to pain or discomfort during the procedure. Tongue pressure, ODK “ta,” ODK “ka,” and chin-tuck force were all found to be statistically significant associations with sarcopenia. Individuals with sarcopenia exhibited lower values of these measures compared with those without sarcopenia in *t*-tests (Table 1). Oral frailty, however, did not differ by sarcopenia status. The crude odds ratio (OR) for sarcopenia in relation to tongue pressure, ODK “ta”, ODK “ka” and chin-tuck force was calculated using univariate logistic regression. The OR for tongue pressure was 0.94 (95% CI: 0.88–1.00), the OR for ODK “ta” was 0.30 (95% CI: 0.17–0.54), the OR for ODK “ka” was 0.50 (95% CI: 0.30–0.84) and the OR for chin-tuck force was 0.64 (95% CI: 0.52–0.78) (Table 2).

To further examine these associations, logistic regression analyses adjusted for age and sex were conducted. For ODK, only the syllable “ta” was included in the analysis because it showed the largest effect size (Cohen’s d) among the ODK measures. In the age- and sex-adjusted model, ODK “ta” and chin-tuck force, excluding tongue pressure, were significantly associated with sarcopenia among the swallowing-related muscle functions. Higher chin-tuck force and greater ODK “ta” were associated with lower odds of sarcopenia (Table 2). Furthermore, exploratory analyses estimated the ORs for tongue pressure, ODK “ta,” and chin-tuck force, including age, sex, BMI, TUG, and TMT Part B as covariates. Only chin-tuck force remained significantly associated with sarcopenia.

### 3.3. Discriminative Abilities of Swallowing-Related Muscle Function for Sarcopenia

**Figure 2:**
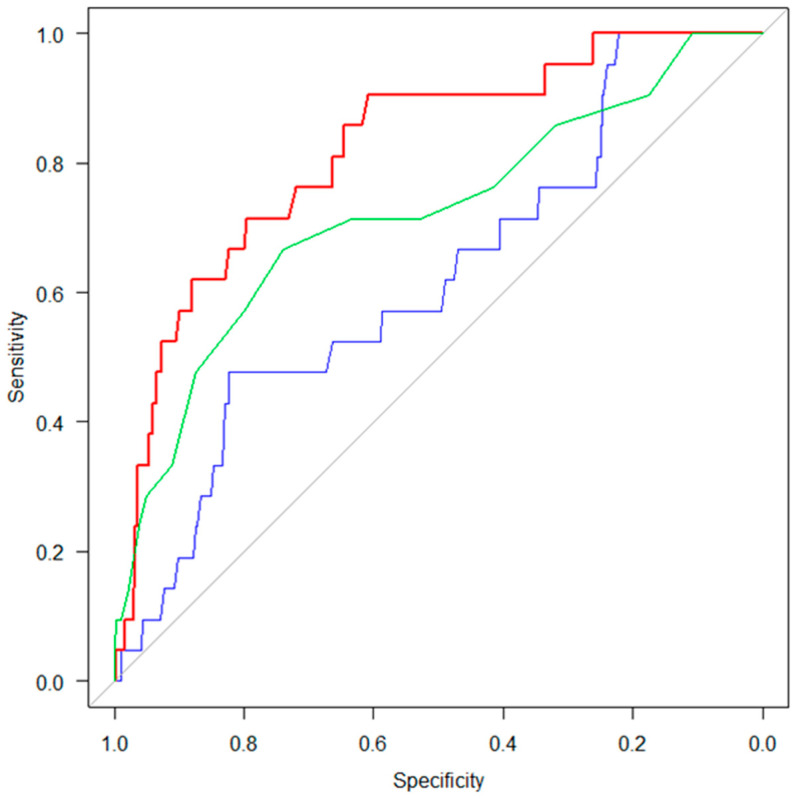
Discriminative abilities of swallowing-related muscle function measures. The figure presents the results of ROC curve analysis for identification of sarcopenia for ODK ‘ta’, tongue pressure and chin-tuck force. The red line represents chin-tuck force, the green line represents ODK “ta”, and the blue line represents tongue pressure. The discriminative ability for sarcopenia of chin-tuck force was significantly superior to that of the tongue pressure.

ROC curve analyses were conducted to assess the discriminative abilities of ODK “ta,” tongue pressure, and chin-tuck force for identifying sarcopenia in the total sample and in sex-stratified subgroups (Table 3). The AUC for chin-tuck force was higher than that for both ODK and tongue pressure in the overall sample, as well as in the gender-specific analyses. Notably, when comparing discriminative performance among the swallowing-related muscle function measures, the AUC for chin-tuck force was significantly larger than that for tongue pressure (Bootstrap method, *p* < 0.001) (Figure 2). The superiority of chin-tuck force over tongue pressure was further supported by NRI and IDI analyses (NRI = 0.893, 95% CI = 0.517–1.270, *p* < 0.001; IDI = 0.086, 95% CI = 0.046–0.127, *p* < 0.001). In contrast, no statistically significant differences were observed between chin-tuck force and ODK “ta” in their discriminative abilities, as indicated by both tests for AUC in the bootstrap method and the NRI and IDI analyses.

## 4. Discussion

In this cross-sectional observational study, we examined the association between swallowing-related cervical muscle function and sarcopenia in community-dwelling older adults. An important strength of the present study is that, in addition to tongue pressure and ODK, which have been widely used in previous research and clinical practice, we assessed force generated during the chin-tuck maneuver (chin-tuck force) as an indicator of swallowing-related cervical muscle function. Our findings showed a statistically significant association between chin-tuck force and sarcopenia, even after adjusting for age and sex. Although the number of participants with sarcopenia was relatively small, exploratory analyses that included BMI and physical function as assessed using the TUG test—both of which are related to sarcopenia—supported the association between chin-tuck force and sarcopenia. Moreover, chin-tuck force demonstrated acceptable discriminatory ability for identifying sarcopenia. Previous studies in related fields have reported associations between tongue pressure or ODK and sarcopenia [15,35]. Consistent with these findings, the present study also found that both tongue pressure and ODK were associated with sarcopenia in univariate analyses. However, few investigations have specifically examined the relationship between chin-tuck force and sarcopenia. Therefore, the findings of this study provide novel insights into the association between swallowing-related cervical musculature and sarcopenia in community-dwelling older adults.

Evidence regarding the association between suprahyoid muscle strength and sarcopenia remains limited. A previous study examined this relationship using jaw-opening force as an index of suprahyoid muscle strength [17] and suggested a potential association with sarcopenia. However, interpretation of its findings was limited by sex-specific inconsistencies and insufficient adjustment for confounding factors. In the present study, an indicator reflecting suprahyoid muscle activity was assessed by measuring the force generated during the chin-tuck maneuver. Previous studies using surface electromyography have demonstrated that the chin-tuck maneuver increases muscle activity in the suprahyoid muscles [36,37]. Moreover, chin-tuck has been reported to be an effective method that enhances suprahyoid muscle activity, while minimizing variability due to differences in task instructions [38]. Kinematic analyses of swallowing have further shown that tucking the chin facilitates vertical displacement of the epiglottis and narrowing of the airway entrance during swallowing [39]. Additionally, chin-tuck against resistance has been identified as an effective therapeutic intervention for improving oropharyngeal dysphagia [25,40]. Taken together, these findings support the validity of measuring force generated during the chin-tuck maneuver as an indicator of swallowing-related cervical muscle function, including that of the suprahyoid muscles.

Regarding the association between swallowing-related muscle function and sarcopenia, the present study found that ODK, tongue pressure, and chin-tuck force were all associated with sarcopenia in univariate analyses. Swallowing function is essential for adequate nutritional intake and involves a variety of swallowing-related muscles, including those related to mastication and occlusion, as well as muscles responsible for laryngeal elevation and descent during swallowing [41]. The results of this study suggest that lower skeletal muscle mass and strength may be associated with various swallowing-related muscle functions, not just those of specific muscles such as the tongue. In contrast, oral frailty was not associated with sarcopenia, even in univariate analyses, in the present study. Previous longitudinal observational studies have reported oral frailty as a predictor of sarcopenia [42]; however, those studies assessed oral frailty using objective measures. The assessment tool used in the present study, the OF-5, although validated, is based on subjective self-reported measures. Subjective assessments of oral function have been reported to be influenced by depressive symptoms [43], which may limit their ability to accurately reflect actual oral function. Therefore, the present findings suggest that, when evaluating swallowing function in relation to sarcopenia, objective assessments of swallowing-related muscle function may provide more reliable information.

Among the swallowing-related muscle function measures assessed in the present study, chin-tuck force showed a significant association with sarcopenia in both the age- and sex-adjusted model and exploratory model. However, the associations for the other measures were attenuated after adjustment. In addition, chin-tuck force demonstrated acceptable discriminatory ability for sarcopenia and showed the highest discriminative performance among the measured variables. These findings should be interpreted with caution, given the limited number of sarcopenia cases. However, the results suggest that sarcopenia in community-dwelling older adults may be more closely associated with swallowing-related cervical muscle function, particularly that involving the suprahyoid muscles, than with other swallowing-related muscle functions. However, the underlying reasons for this observation cannot be clearly determined based on the results of this study alone. One possible explanation may relate to functional differences during swallowing between the tongue, which is assessed by tongue pressure and ODK, and the suprahyoid muscles. The tongue plays a primary role in propelling the bolus into the pharynx during the oral phase of swallowing, whereas the suprahyoid muscles contribute to laryngeal elevation and closure of the airway entrance during the pharyngeal phase [41]. The suprahyoid muscles have been reported to be affected by age-related changes, and diminished activity of these muscles is considered one of the mechanisms responsible for aspiration in older adults [20,21,22]. Moreover, a decrease in upper esophageal sphincter opening diameter and prolongation of pharyngeal clearance time—both of which involve suprahyoid muscle function—have been documented in older adults [44,45]. In contrast, tongue function has been reported to be influenced by factors other than swallowing, such as social interaction and opportunities for vocalization in daily life [46,47]. Taken together, these findings suggest that suprahyoid muscle function may more sensitively reflect swallowing dysfunction in older adults and may be more closely associated with sarcopenia, potentially through pathways related to undernutrition. Nevertheless, further research is needed to elucidate the mechanisms underlying the association between suprahyoid muscle function and sarcopenia. At the very least, the present findings emphasize the importance of comprehensive and objective assessment of swallowing-related muscle function in older adults, including evaluation of suprahyoid muscle function.

Several limitations of this study need to be acknowledged. First, the cross-sectional design precludes any inference regarding causal relationships between swallowing-related muscle function and sarcopenia. It is, therefore, not possible to determine whether impairments in swallowing-related muscle function contribute to the development of sarcopenia or whether sarcopenia itself leads to a decline in swallowing-related muscle function. In other words, reverse causation cannot be ruled out, as sarcopenia may affect swallowing-related cervical muscle function. Second, the study population consisted exclusively of community-dwelling older adults; thus, generalizability of the findings to frailer populations, such as institutionalized individuals or those requiring long-term care, may be limited. Additionally, the findings of this study do not apply to patients with bulbar palsy, including those with neurodegenerative diseases. This is because the pathological mechanisms of swallowing disorders differ from sarcopenia-related swallowing dysfunction in older adults. Third, the small number of participants with sarcopenia may have affected the results of the ROC curve analysis and the stability of the multivariable models. In particular, the AUC estimates may be subject to optimism bias due to the limited number of events. We conducted additional exploratory analyses with multiple covariates; however, these results should be interpreted with caution due to the potential for overfitting. Therefore, findings from minimally adjusted models may be more reliable. Further studies with larger sample sizes are needed to confirm these results. Fourth, although the chin-tuck maneuver has been shown to strongly activate the suprahyoid muscles, the force measured during this task may not exclusively reflect suprahyoid muscle strength, because other cervical flexor muscles may also contribute to the generated force. Therefore, the measured force should not be interpreted as an isolated measure of suprahyoid muscle strength. In addition, direct assessments of muscle morphology or muscle mass using imaging techniques were not performed. Fifth, despite adjustment for multiple potential confounders, residual confounding by unmeasured factors, such as detailed nutritional intake, inflammatory status, and physical activity levels, cannot be ruled out. In particular, inflammatory markers and physical activity levels were not assessed due to limitations in research staff and available resources. Since these factors may be associated with sarcopenia, future studies that incorporate these variables could provide a more comprehensive understanding of the relationship between swallowing-related cervical muscle function and sarcopenia. Sixth, swallowing function was evaluated primarily through muscle function measurements rather than comprehensive instrumental swallowing assessments, such as videofluoroscopic or endoscopic examinations. Additionally, since an assessment of oral function was performed using a self-rating method, the results may be inaccurate. Finally, because our research group developed the device used to measure chin-tuck force, the possibility of potential bias cannot be completely excluded. However, to maintain objectivity and fairness in the study, swallowing-related muscle function was also evaluated using well-established measures, such as tongue pressure and ODK. Further independent studies by research groups not involved in developing the device are needed to confirm the generalizability and external validity of these findings. These limitations highlight the need for future longitudinal and interventional studies to clarify the temporal relationships and underlying mechanisms linking swallowing-related muscle function, particularly suprahyoid muscle function, with sarcopenia in older adults. The results of this study suggest that lower skeletal muscle mass and strength may be associated not only with specific muscles, such as the tongue, but also with other swallowing-related muscle function. Furthermore, unlike handgrip strength, which is used to diagnose sarcopenia, chin-tuck force reflects swallowing-related cervical muscle function. Moreover, because the chin-tuck force assessment is non-invasive and relatively simple, it may be feasible to incorporate this measurement into the evaluation of swallowing-related muscle function in primary care settings. This may provide additional information to help understand sarcopenia-related swallowing dysfunction in older adults.

## 5. Conclusions

This study examined the association between sarcopenia and swallowing-related muscle function, including chin-tuck force, tongue pressure, and ODK, in community-dwelling older adults. Even after adjusting for age and sex, the force generated during the chin-tuck maneuver was found to have a statistically significant association with sarcopenia. In addition, the chin-tuck force demonstrated acceptable discriminatory ability for sarcopenia and showed the highest discriminative performance among the swallowing-related muscle function measures evaluated. These findings suggest that, when assessing swallowing-related muscle function in relation to sarcopenia among community-dwelling older adults, a comprehensive and objective evaluation that includes suprahyoid muscle function may provide useful information.

## 6. Patents

Dr. Kamide and Dr. Murakami are the inventors of the measuring device for chin-tuck force (Patent No. 7495133), which is registered with the Japan Patent Office.

## Figures and Tables

**Table 1:** Descriptive statistics in the total sample and in samples stratified by sarcopenia.

	Total Sample		Non Sarcopenia		Sarcopenia		Between Group *p* Value
	n = 390		n = 369		n = 21		
	Mean/n	SD/%	Mean/n	SD/%	Mean/n	SD/%	
Age (years)	74.8	5.4	74.6	5.3	78.5	6.5	0.001
Sex (female)	276	70.8	260	70.5	16	76.2	0.805
Body mass index (kg/m^2^)	22.3	2.9	22.4	2.8	20.4	3.0	0.001
Hypertension	135	34.6	132	35.8	3	14.3	0.058
Diabetes mellitus	36	9.2	32	8.7	4	19.0	0.117
Stroke	7	1.8	6	1.6	1	4.8	0.323
Kidney disease	11	2.8	10	2.7	1	4.8	0.46
Respiratory disease	13	3.3	12	3.3	1	4.8	0.519
Heart disease	22	5.6	22	6.0	0	0	0.621
Polypharmacy	26	6.7	26	7.1	0	0	0.382
TMIG-IC ^1^ score (points)	11.9	1.3	11.9	1.3	11.4	1.4	0.112
Depressive symptoms	69	17.7	67	18.2	2	9.5	0.394
Living alone	64	16.4	58	18.8	6	35.3	0.115
Intake of dairy products (every day)	362	92.8	344	93.5	18	85.7	0.171
Intake of legumes or eggs (≥2/w)	379	97.2	358	97.0	21	100.0	>0.99
Intake of meat, fish (every day)	351	90.0	331	89.9	20	95.2	0.708
Timed Up and Go test (s)	6	1.1	5.9	1.0	7.1	1.4	<0.001
Grip strength (kg)	26.8	7.4	27.2	7.3	18.5	4.4	<0.001
Low muscle strength	35	9.0	14	3.8	21	100.0	<0.001
SMI ^2^ (kg/m^2^)	6.5	0.9	6.5	0.9	5.5	0.7	<0.001
Low muscle mass	104	26.7	83	22.5	21	100.0	<0.001
Trail Making test Part A (s)	54.5	18.3	54.2	18.3	61.0	16.2	0.094
Trail Making test Part B (s)	98.0	42.1	96.5	41.1	124.3	52.3	0.003
Oral frailty	156	40.0	146	39.8	10	47.6	0.499
Tongue pressure (kPa)	31.8	7.5	32.0	7.6	28.7	6.6	0.049
ODK ^3^, “pa” (times/s)	6.5	0.7	6.6	0.7	6.3	0.7	0.054
ODK ^3^, “ta” (times/s)	6.5	0.7	6.5	0.7	5.8	0.9	<0.001
ODK ^3^, “ka” (times/s)	6.0	0.7	6	0.7	5.5	1.0	0.007
Chin-tuck force (kg)	11.5	4.1	11.7	4.0	7.6	2.5	<0.001

^1^ TMIG-IC: Tokyo Metropolitan Institute of Gerontology Index of Competence, ^2^ SMI: Skeletal muscle mass index, ^3^ ODK: Oral diadochokinesis.

**Table 2:** Association between swallowing-related muscle function and sarcopenia.

	Crude Models		Age and Sex Adjusted Models		Exploratory Analysis Models	
	OR ^2^ (95% CI) ^3^	*p* Value	OR ^2^ (95% CI) ^3^	*p* Value	OR ^2^ (95% CI) ^3^	*p* Value
ODK ^1^, “ta” (times/s)	0.30 (0.17–0.54)	<0.001	0.34 (0.18–0.62)	<0.001	0.55 (0.27–1.12)	0.097
Tongue pressure (kPa)	0.94 (0.88–1.00)	0.049	0.96 (0.90–1.02)	0.464	0.98 (0.90–1.06)	0.560
Chin-tuck force (kg)	0.64 (0.52–0.78)	<0.001	0.59 (0.47–0.73)	<0.001	0.65 (0.50–0.84)	<0.001

^1^ ODK: Oral diadochokinesis, ^2^ OR: odds ratio, ^3^ 95% CI: 95% confidence interval. The highest variance inflation factor value for the independent variables was 1.78 among all models. The exploratory model included age, sex, body mass index, the Timed Up and Go test, and the Trail Making test.

**Table 3:** Discriminative abilities of swallowing-related muscle function.

	Sample	AUC ^2^	95% CI ^3^
ODK ^1^, “ta” (times/s)	Total	0.72	0.59–0.84
	Male	0.76	0.51–0.96
	Female	0.71	0.56–0.85
Tongue pressure (kPa)	Total	0.62	0.50–0.74
	Male	0.49	0.22–0.76
	Female	0.66	0.54–0.79
Chin-tuck force (kg)	Total	0.82	0.72–0.90
	Male	0.88	0.77–0.97
	Female	0.86	0.77–0.92

^1^ ODK: Oral diadochokinesis, ^2^ AUC: area under the curve, ^3^ 95% CI: 95% confidence interval.

## Data Availability

The datasets presented in this article are not readily available because the data are part of an ongoing study. Requests to access the datasets should be directed to the corresponding author.

## Abbreviations

The following abbreviations are used in this manuscript:

ASM	Appendicular skeletal muscle mass
AUC	Area under the curve
AWGS	Asian Working Group for Sarcopenia
BMI	Body mass index
IADL	Instrumental activities of daily living
IDI	Integrated discrimination improvement
NRI	Net reclassification improvement
ODK	Oral diadochokinesis
OF-5	Oral Frailty Five-Item Checklist
OR	Odds ratio
ROC	Receiver operating characteristic
SMI	Skeletal muscle mass index
TMIG-IC	Tokyo Metropolitan Institute of Gerontology Index of Competence
TMT	Trail Making Test
TUG	Timed Up and Go
